# The Effects of Healthy Aging on Right Ventricular Structure and Biomechanical Properties: A Pilot Study

**DOI:** 10.3389/fmed.2021.751338

**Published:** 2022-01-10

**Authors:** Danial Sharifi Kia, Yuanjun Shen, Timothy N. Bachman, Elena A. Goncharova, Kang Kim, Marc A. Simon

**Affiliations:** ^1^Department of Bioengineering, University of Pittsburgh, Pittsburgh, PA, United States; ^2^Pittsburgh Heart, Lung, Blood and Vascular Medicine Institute, University of Pittsburgh and University of Pittsburgh Medical Center, Pittsburgh, PA, United States; ^3^Davis School of Medicine Lung Center, University of California, Davis, Davis, CA, United States; ^4^Division of Pulmonary, Allergy and Critical Care Medicine, School of Medicine, University of Pittsburgh, Pittsburgh, PA, United States; ^5^Heart and Vascular Institute, University of Pittsburgh Medical Center, Pittsburgh, PA, United States; ^6^Division of Cardiology, School of Medicine, University of Pittsburgh, Pittsburgh, PA, United States; ^7^McGowan Institute for Regenerative Medicine, University of Pittsburgh, Pittsburgh, PA, United States; ^8^Department of Mechanical Engineering and Materials Science, University of Pittsburgh, Pittsburgh, PA, United States; ^9^Center for Ultrasound Molecular Imaging and Therapeutics, University of Pittsburgh, Pittsburgh, PA, United States; ^10^Division of Cardiology, Department of Medicine, University of California, San Francisco, San Francisco, CA, United States

**Keywords:** right ventricular remodeling, aging, right ventricular biomechanics, ventricular structure, hemodynamics

## Abstract

Healthy aging has been associated with alterations in pulmonary vascular and right ventricular (RV) hemodynamics, potentially leading to RV remodeling. Despite the current evidence suggesting an association between aging and alterations in RV function and higher prevalence of pulmonary hypertension in the elderly, limited data exist on age-related differences in RV structure and biomechanics. In this work, we report our preliminary findings on the effects of healthy aging on RV structure, function, and biomechanical properties. Hemodynamic measurements, biaxial mechanical testing, constitutive modeling, and quantitative transmural histological analysis were employed to study two groups of male Sprague-Dawley rats: control (11 weeks) and aging (80 weeks). Aging was associated with increases in RV peak pressures (+17%, *p* = 0.017), RV contractility (+52%, *p* = 0.004), and RV wall thickness (+38%, *p* = 0.001). Longitudinal realignment of RV collagen (16.4°, *p* = 0.013) and myofibers (14.6°, *p* = 0.017) were observed with aging, accompanied by transmural cardiomyocyte loss and fibrosis. Aging led to increased RV myofiber stiffness (+141%, *p* = 0.003), in addition to a bimodal alteration in the biaxial biomechanical properties of the RV free wall, resulting in increased tissue-level stiffness in the low-strain region, while progressing into decreased stiffness at higher strains. Our results demonstrate that healthy aging may modulate RV remodeling via increased peak pressures, cardiomyocyte loss, fibrosis, fiber reorientation, and altered mechanical properties in male Sprague-Dawley rats. Similarities were observed between aging-induced remodeling patterns and those of RV remodeling in pressure overload. These findings may help our understanding of age-related changes in the cardiovascular fitness and response to disease.

## Introduction

Healthy aging is associated with alterations in right ventricular (RV) structure and function in subjects with no underlying cardiopulmonary disease (1–5). Aging has been shown to result in pulmonary artery (PA) remodeling (6, 7) and increased pulmonary vascular resistance (5, 8). Echocardiographic studies on RV function have found a strong positive correlation between aging and PA systolic pressures (9, 10). This, in turn, may lead to increased RV afterload and RV remodeling (11, 12), altered contraction dynamics (13), and decreased global and segmental RV systolic strains (14). Previous work has demonstrated that healthy aging results in diminished RV hypertrophy in response to pressure overload (11, 15). Age-related differences exist in the survival rates of pulmonary hypertension (PH) patients in which older patients show more severe characteristics with poor response to therapeutic interventions (16, 17).

In recent years, biomechanical analysis techniques have been employed to better understand the underlying mechanisms of RV remodeling (18–21) and have closely linked RV biomechanics to physiological function (22). Despite the evidence suggesting an association between aging and alterations in RV structure/function, the literature has focused on younger animal models and limited data exist on age-associated differences in RV biomechanics. Similar to RV adaption to pressure overload in PH, alterations in PA resistance and systolic pressures with healthy aging have the potential to trigger RV remodeling, leading to altered organ, tissue, and fiber-level biomechanics.

In this work, we present our pilot findings on the effects of healthy aging on RV biomechanical properties. Our study provides preliminary insights into how healthy aging may modulate RV remodeling and lays the groundwork for future studies to further evaluate the age-related differences in RV response to pressure overload.

## Methods

**Figure 1:**
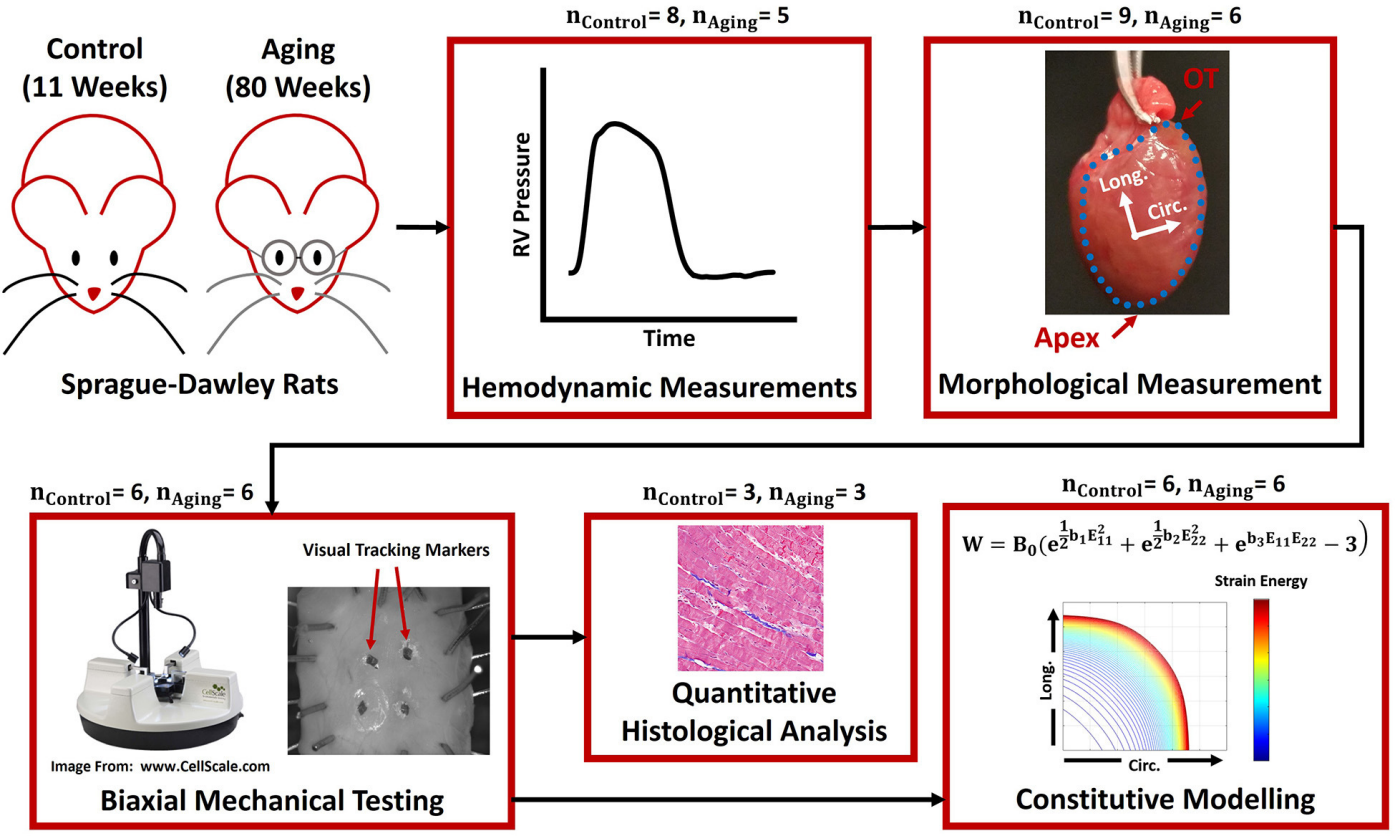
The framework used to study RV remodeling with healthy aging. *In vivo* terminal invasive hemodynamic measurements were performed on young controls and aging Sprague-Dawley rats, followed by harvesting the heart and morphological measurements, biaxial mechanical testing, constitutive modeling, and quantitative transmural histological analysis. The blue dots in the “Morphological Measurement” panel demonstrate the boundaries of the RV free wall, while the Circ-Long coordinate system indicates the orientation of square specimens harvested for mechanical testing. RV, Right ventricle; OT, Outflow tract; Circ, Circumferential; Long, Longitudinal.

The data acquired during this study are available from the corresponding author on reasonable request. A total of 15 male Sprague-Dawley rats corresponding to young (controls, ~11 weeks, weighing 327 ± 9 g, *n*_*Control*_ = 9) and old (~80 weeks, weighing 789 ± 3 g, *n*_*Aging*_ = 6) age groups were studied using a multi-scale biomechanical analysis framework. Historical data from a recent study in our laboratory (18) was used for the control animals in this work. An ~70-week age difference was considered sufficient to study the effects of healthy aging on RV structure/function in the absence of pathological events arising with senescence in older animals, previously reported to begin at ~85 weeks in rats (23). The young and old rats in this work correspond to ~15 and 55 years in human age, respectively (23). 11-week old rats were chosen for our control group to facilitate comparison of our findings on the effects of aging with previous work on RV biomechanics in murine models, which typically utilize rats of this age (19, 24, 25). Figure 1 summarizes the experimental procedures and analysis techniques used in this study. As further demonstrated in Supplementary Figure 1, *n* = 6 animals were dedicated to each group to study the effects of aging on RV hemodynamics, morphology, and biomechanical properties. Histological analysis for the aging cohort, was performed on a sub-set (*n* = 3) of the 6 animals used for hemodynamics and biomechanical analysis, mainly due to the limited availability of aging animals. In the control group, however, we were able to have 3 separate animals dedicated to histological analysis. Hemodynamic and morphological measurements were performed on these 3 additional control animals, in order to confirm normal RV function. All animal procedures were approved by University of Pittsburgh's IACUC (protocol# 18113872 and 19126652).

### Hemodynamic and Morphological Measurements

Using standard techniques (19), *in-vivo* terminal invasive pressure catheterization was performed on both groups (*n*_*Control*_ = 8; *n*_*Aging*_ = 5). Open-chest hemodynamic measurements were performed under anesthesia induced via inhalation of isoflurane, while the animals were placed on a heated table (37°C) and monitored using a rectal probe. Pressure waveforms were then acquired using a conductance catheter and analyzed for common hemodynamic metrics of RV function. Due to lack of cuvette calibration of catheters for conversion of volume measurements from relative-volume-units to absolute measurements, only pressure-based hemodynamic data are reported and compared in the current work. RV heart rate was calculated by evaluating the periodicity of the waveforms (peak-to-peak time). Peak pressures were characterized as the maximum pressure experienced by the RV during a cardiac cycle (P_max_), while end-diastolic pressures were identified as pressures at the point of the maximum second derivative of the waveforms, $\left(\frac{d^{2}p}{dt^{2}}\right)\max$. Load-dependent measures of RV contractility and relaxation were, respectively, calculated by evaluating the maximum and minimum of the time derivatives of pressure waveforms ($\frac{dp}{dt}$max and $\frac{dp}{dt}$min). Contractility index was then obtained using the ratio of $\frac{dp}{dt}$max over the maximum pressure experienced by the RV over a cardiac cycle (RV peak pressure; P_max_). Additionally, the time-constant of RV relaxation (tau) was calculated as (26):


(1) $$\begin{array}{l} \ln\;\left(P\right)\;=\;-\frac{1}{\tau }\cdot t\;+\;B \end{array}$$

Where *P* represents the RV pressure waveform beginning at $\frac{dp}{dt}$min until the minimum RV pressure experienced during a cardiac cycle (P_min_), B is an intercept, *t* represents the time during a cardiac cycle, and τ (tau) is the time-constant of RV relaxation, measured via linear regression of equation 1 to the acquired pressure waveforms in MATLAB (Mathworks, Natick, MA). Hemodynamic parameters for each animal were evaluated using average measurements from at least three consecutive cycles, except for tau, which was obtained from a single beat (due to limitations of our custom hemodynamic analysis subroutines for estimation of tau).

Following hemodynamic measurements, the heart was harvested and arrested by placement in cardioplegic solution (27). Subsequently, the RV free wall (RVFW) was dissected and measurements were acquired for the Fulton index [ratio of RV weight to weight of the left ventricle (LV) + intraventricular septum] and RVFW thickness (*n*_*Control*_ = 9, *n*_*Aging*_ = 6). All measurements were performed in air, at room temperature (23°C), using a scale (0.1 mg readability; Mettler-Toledo International Inc., Columbus, OH) and a thickness gauge (0.025 mm precision; L. S. Starrett Company, Athol, MA).

### Biomechanical Characterization

Following morphological measurements, square specimens with a circumferential-longitudinal orientation (Figure 1) were harvested from the RVFW to undergo biaxial mechanical testing (*n*_*Control*_ = 6, *n*_*Aging*_ = 6). Specimens were mounted on a BioTester testing device (CellScale, Waterloo, ON, Canada), using a suture and pulley mechanism for minimal shear loading (28). Samples were then submerged in modified Krebs solution with 2,3-Butanedione monoxime and oxygen to ensure tissue viability (27). Our previous work (19) has shown that this media bath can effectively maintain tissue viability up to 90 min, via passive diffusion. All measurements in this study were concluded within 45–80 min of harvesting the heart.

RVFW mechanical properties were characterized using multi-protocol displacement-controlled biaxial loading scenarios (1:1, 1:2, 2:1, 1:4, 4:1, 1:6, and 6:1 displacement ratios). Previous work has demonstrated that this loading protocol can effectively capture the biaxial RVFW properties under a wide range of possible strains (18, 27, 29), generating adequate data for parameter characterization of constitutive models. Each specimen underwent 15 cycles of 1:1 displacement-controlled preconditioning, before the start of data acquisition. Four visual tracking markers were placed on the epicardial surface of the RVFW specimens and marker displacements (recorded using a CCD camera) were post-processed via standard techniques (27, 29, 30) to obtain the deformation gradient tensor (F), using a four-node finite-element approximation (31). Components of the Green–Lagrange strain tensor (E) were then calculated as $E=\frac{1}{2}\left(F^{T}F-I\right)$, where I is the identity tensor. Biaxial force measurements and initial specimen dimensions were used to obtain the 1st Piola-Kirchhoff stress tensor (*P*) by calculating the ratio of forces in the deformed configuration over the cross-sectional area in the reference configuration. The 2nd Piola-Kirchhoff stress tensor (S) was then evaluated as *S* = *F*^−1^*P*. Stress-strain data was post-processed under a plane-stress approximation, using a finite deformation analysis framework in Mathcad (PTC, Needham, MA).

Using previously established techniques (19, 32), equibiaxial strain-controlled responses of RVFW specimens were interpolated from the acquired multi-protocol displacement-controlled experimental data via biharmonic spline interpolations in MATLAB (Supplementary Figure 2). As previously discussed, equibiaxial strain-controlled responses are accompanied by unique tissue kinematics with no fiber rotations (33) and, therefore, could be used to estimate fiber-level mechanical properties from tissue-level measurements, independent of fiber orientation and splay (19, 21, 32). Effective fiber-ensemble (EFE) stresses, representing the fiber-level response of combined collagen and myofiber bundles, were then estimated from tissue-level measurements as (19, 32):


(2) $$\begin{array}{l} {\text{S}}_{\text{EFE}}={\left({\text{S}}_{11}\right)}_{\text{Equibiaxial}}+{\left({\text{S}}_{22}\right)}_{\text{Equibiaxial}} \end{array}$$

Here, S_EFE_ represents the EFE stress of the combined collagen-myofiber bundles, and (_S_11_)Equibiaxial_ and (_S_22_)Equibiaxial_ are the interpolated biaxial tissue-level 2nd Piola-Kirchhoff stresses under equibiaxial strains, respectively, in the circumferential and longitudinal directions. We assumed the initial nearly-linear, low-strain portion of the EFE stress-strain responses to be mostly dominated by myofibers, while collagen fibers dominated the high-strain response following recruitment (18, 34) (Supplementary Figure 3). To categorize the data before and after collagen recruitment, equation 2 was differentiated with respect to EFE strain (E_EFE_), to evaluate the changes in EFE stiffness ($\text{T}{\text{M}}_{\text{EFE}}=\frac{\partial {\text{S}}_{\text{EFE}}}{\partial {\text{E}}_{\text{EFE}}}$; where TM_EFE_ is the EFE stiffness). For specimens in both groups, we observed a relatively constant-stiffness region (relatively linear stress-strain behavior, dominated by myofibers), followed by beginning of collagen recruitment and an abrupt increase in EFE stiffness (Supplementary Figure 4). The strain at which collagen fibers begin recruitment was defined as the point where there is a significant elevation in EFE stiffness compared to the stiffness trends prior to that point. This was quantified as the point where TM_EFE_ (EFE stiffness) was significantly elevated outside of the *Z* = 4.417 confidence interval of the distribution of TM_EFE_ measurements before that point. A Z-value of 4.417 (99.999% confidence interval) was chosen as a threshold for maximal confidence in the detected increase in stiffness, avoiding false detection of collagen recruitment strain due to potential fluctuations in the low-strain data, resulting from data acquisition noise. The EFE strain at the *n* + 1th point of the EFE stiffness-strain plot (Supplementary Figure 4) was defined as the collagen recruitment strain, if:


(3) $$\begin{array}{l} {\left({\text{TM}}_{\text{EFE}}\right)}_{\text{n}+1}>\frac{1}{\text{n}}\sum_{1}^{\text{n}}{\left({\text{TM}}_{\text{EFE}}\right)}_{\text{n}}\\ \;+\;4.417\;*\;\frac{\sqrt{\frac{\sum_{1}^{\text{n}}{\left[{\left({\text{TM}}_{\text{EFE}}\right)}_{\text{n}}-\frac{1}{\text{n}}\sum_{1}^{\text{n}}{\left({\text{TM}}_{\text{EFE}}\right)}_{\text{n}}\right]}^{2}}{\text{n}-1}}}{\sqrt{\text{n}}} \end{array}$$

Here, (TM_EFE_)_*n*+1_ is the EFE stiffness at the *n* + 1th point of the EFE stiffness-strain data (Supplementary Figure 4). The right-hand side of the inequality represents the upper bound of the TM_EFE_ confidence interval based on the TM_EFE_ data up to the n^th^ point. The beginning of collagen recruitment was defined as the first point where the inequality in equation 3 is satisfied. The EFE stress-strain data before collagen recruitment was then used for myofiber stiffness estimations, using a rule-of-mixtures approach (18, 19, 35):


(4) $$\begin{array}{l} {\left(\text{T}{\text{M}}_{\text{EFE}}\right)}_{\text{Before Collagen Recruitment}}=\;\phi_{\text{Myofiber}}\text{T}{\text{M}}_{\text{Myofiber}}\\ \;+\phi_{\text{Collagen}}\text{T}{\text{M}}_{\text{Collagen}} \end{array}$$

Where (T_M_EFE_)Before Collagen Recruitment_ is the slope of the line fitted to the initial low-strain portion of the EFE stress-strain curve (Supplementary Figure 5), ϕ_Myofiber_ and ϕ_Collagen_ represent the myofiber and collagen area fractions in RVFW specimens (measures of tissue content; acquired from histological measurements), and TM_Myofiber_ and TM_Collagen_ are the effective myofiber and collagen stiffnesses, respectively. Assuming the initial portion of the stress-strain data to be dominated by myofibers (minimal collagen recruitment, TM_Collagen_ = 0), effective myofiber stiffness for each specimen was estimated as:


(5) $$\begin{array}{l} \text{T}{\text{M}}_{\text{Myofiber}}=\;\frac{{\left(\text{T}{\text{M}}_{\text{EFE}}\right)}_{\text{Before Collagen Recruitment}}}{\phi_{\text{Myofiber}}} \end{array}$$

Additionally, a non-linear anisotropic constitutive model (36) was used to model the response of the RVFW specimens in each cohort:


(6) $$\begin{array}{l} \text{W}={\text{B}}_{0}\left({\text{e}}^{\frac{1}{2}{\text{b}}_{1}{\text{E}}_{11}^{2}}+{\text{e}}^{\frac{1}{2}{\text{b}}_{2}{\text{E}}_{22}^{2}}+{\text{e}}^{{\text{b}}_{3}{\text{E}}_{11}{\text{E}}_{22}}-3\right) \end{array}$$

Here, W is the strain energy density, E_11_ and E_22_, respectively, represent the circumferential and longitudinal (apex-to-base) Green-Lagrange strains, B_0_ is a scaling factor and b_1_, b_2_ and b_3_ are metrics for the circumferential, longitudinal and in-plane coupling stiffness of the RVFW, respectively (19). 2nd Piola–Kirchhoff stress components were obtained by differentiating equation 6 with respect to Green-Lagrange strain:


(7) $$\begin{array}{l} {\left({\text{S}}_{11}\right)}_{\text{Model}-\text{Predicted}}=\frac{\partial \text{W}}{\partial {\text{E}}_{11}}={\text{B}}_{0}({\text{b}}_{1}{\text{E}}_{11}{\text{e}}^{\frac{1}{2}{\text{b}}_{1}{\text{E}}_{11}^{2}}+{\text{b}}_{3}{\text{E}}_{22}{\text{e}}^{{\text{b}}_{3}{\text{E}}_{11}{\text{E}}_{22}})\\ {\left({\text{S}}_{22}\right)}_{\text{Model}-\text{Predicted}}=\frac{\partial \text{W}}{\partial {\text{E}}_{22}}={\text{B}}_{0}({\text{b}}_{2}{\text{E}}_{22}{\text{e}}^{\frac{1}{2}{\text{b}}_{2}{\text{E}}_{22}^{\text{2}}}+{\text{b}}_{3}{\text{E}}_{11}{\text{e}}^{{\text{b}}_{3}{\text{E}}_{11}{\text{E}}_{22}}) \end{array}$$

Where (S_11_)_Model−Predicted_ and (S_22_)_Model−Predicted_ are the model-predicted stress components in the circumferential and longitudinal directions, respectively. Using equation 7 and the acquired multi-protocol experimental stress-strain data, model parameters were estimated for each specimen using a trust-region-reflective non-linear least-squares optimization algorithm in MATLAB, to minimize the difference between model-predicted and experimentally acquired data. A R^2^ measure was used to evaluate the goodness of fit. Cohort-specific strain energy maps in the low-strain and high-strain regions were then generated by taking the average of all strain energy distributions in the circumferential-longitudinal strain space for specimens in each cohort, facilitating holistic model-based evaluation of RVFW biomechanical properties over a wide range of loading scenarios.

### Quantitative Histological Analysis

Transmural histological staining was performed on a sub-group of specimens (n_Control_ = 3, n_Aging_ = 3) to quantify the effects of aging on RV fiber architecture. Sample sizes were chosen based on our previous work showing minimal between-sample variabilities in RV content and fiber architecture (18, 19). Specimen fixation was carried out using 10% neutral buffered formalin followed by staining of RVFW specimens using Masson's trichrome, resulting in collagen fibers stained in blue and myofibers in red/pink. A total of 11–17 sections were obtained for each specimen, from epi to endocardium, at 50–75 μm increments. Transmural area fractions of collagen and myofibers were then quantified via manual RGB-based thresholding of the histological images (collagen: blue, myofibers: red/pink) to analyze the effects of aging on RVFW composition. Area fractions were calculated as the ratio of the area occupied by respective blue/red pixels, divided by the total area within the region of interest. In addition, cardiomyocyte width was measured from the histological data to investigate the role of aging in RV hypertrophy (40 measurements performed on each specimen at different sites along the myofibers). Furthermore, similar to previous work (18), the orientation of RVFW collagen and myofibers and the coherency of collagen fiber distributions were quantified transmurally, using gradient-based image analysis techniques (37). Following segmentation of histological sections based on the appropriate RGB threshold, local image gradients at each section were used to construct the structure tensor of the gradient map in order to analyze the transmural orientation of RVFW collagen and myofibers (18, 19, 37):


(8) $$\begin{array}{l} \text{T}=\left[\begin{array}{cc} \iint \text{R}\left(\text{x},\text{y}\right){\text{I}}_{\text{x}}\left(\text{x},\text{y}\right){\text{I}}_{\text{x}}\left(\text{x},\text{y}\right)\text{dxdy} & \iint \text{R}\left(\text{x},\text{y}\right){\text{I}}_{\text{x}}\left(\text{x},\text{y}\right){\text{I}}_{\text{y}}\left(\text{x},\text{y}\right)\text{dxdy}\\ \iint \text{R}\left(\text{x},\text{y}\right){\text{I}}_{\text{x}}\left(\text{x},\text{y}\right){\text{I}}_{\text{y}}\left(\text{x},\text{y}\right)\text{dxdy} & \iint \text{R}\left(\text{x},\text{y}\right){\text{I}}_{\text{y}}\left(\text{x},\text{y}\right){\text{I}}_{\text{y}}\left(\text{x},\text{y}\right)\text{dxdy}\\ \; \end{array}\right] \end{array}$$

Here, T is the symmetric positive-definite structure tensor, R(x,y) is a gaussian weighting function which specifies the integration region of interest (37), and I_x_ and I_y_ are the partial spatial derivatives of the histological image (I), respectively, in x and y directions. The 1st eigen vector of T indicates the dominant fiber orientation at each histological section (19, 37). For all data presented in this work, 0° corresponds to the circumferential direction, while +90° points toward the apex-to-base (longitudinal) direction. Moreover, using the eigen values of the structure tensor in equation 8, collagen fiber coherency was evaluated as:


(9) $$\begin{array}{l} \text{C}=\;\frac{\lambda_{1}-\lambda_{2}}{\lambda_{1}+\lambda_{2}}\times 100 \end{array}$$

where λ_1_ and λ_2_ correspond to the 1st and 2nd eigen values of the structure tensor T (37). 0% collagen fiber coherency corresponds to a sparse (non-coherent), randomly distributed fiber architecture, while 100% coherency indicates a highly-aligned, tightly packed, continuous (coherent) collagen fiber distribution (38).

A total of 66 histological sections were analyzed for the control and aging groups. We performed linear interpolations to report the histological data on an equally-spaced grid, against normalized tissue thickness (0–100% thickness). In case of data categorization (Epi, Mid and Endo groups), the data between 0 and 20% thickness were used for the epicardium, while the data between 80 and 100% thickness correspond to the endocardium. Orientation analysis and image segmentation were performed using the OrientationJ toolbox (37, 39) in ImageJ (imagej.nih.gov).

### Statistical Analysis

Data are presented with mean ± standard error of the mean. Sample normality and homogeneity of variances were assessed using the Shapiro–Wilk test and Bartlett's test of homoscedasticity. Circular statistics was employed for fiber orientation analysis, using the Watson–Williams test in the CircStat toolbox (40) in MATLAB. For all other data, in case of normality and homoscedasticity, a two-sided unpaired student's *t*-test was used for statistical comparisons, while non-normal distributions were compared using Mann–Whitney *U*-tests. For all purposes, *p* < 0.05 was considered statistically significant. Statistical analyses were performed in the R software package (R Foundation for Statistical Computing, Vienna, Austria).

## Results

### RV Hemodynamics and Morphology

**Figure 2:**
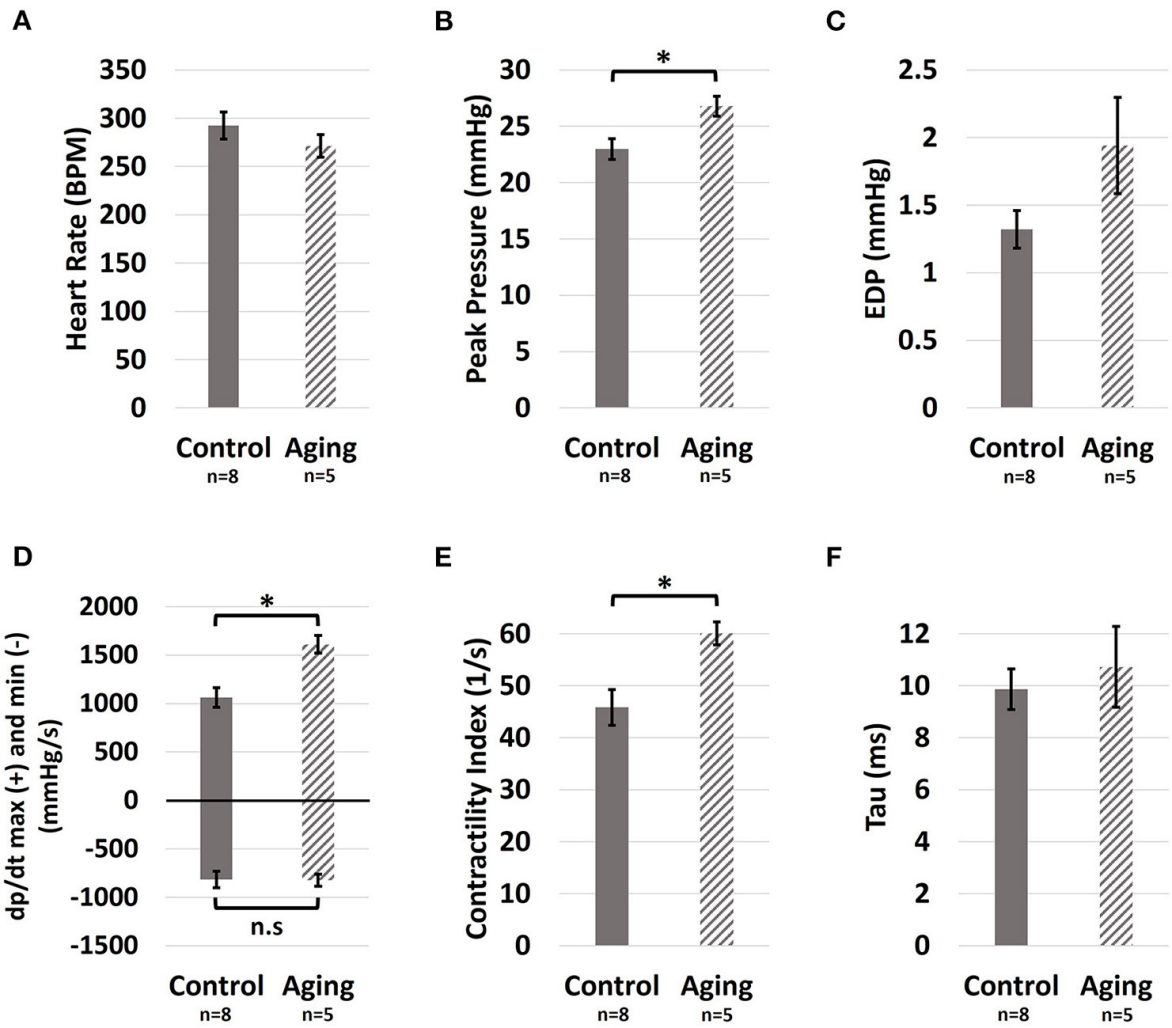
Hemodynamic measures of the effects of healthy aging on RV **(A)** Heart rate, **(B)** Peak pressure, **(C)** End-diastolic pressure, **(D)** $\frac{dp}{dt}$max (positive side) and $\frac{dp}{dt}$min (negative side), **(E)** Contractility index, and **(F)** The preload-independent measure of relaxation (tau). Healthy aging significantly increased RV peak pressures and the load-dependent measures of RV contractility ($\frac{dp}{dt}$max and contractility index), while not affecting the heart rate, end-diastolic pressures (EDP), and relaxation function ($\frac{dp}{dt}$min and tau). Error bars represent standard error of the mean (SEM). ^*^Indicates *p* < 0.05. RV, Right ventricle; BPM, Beats per minute; EDP, End-diastolic pressure; $\frac{dp}{dt}$max and min, Load-dependent measures of RV contractility and relaxation; n.s, Non-significant.

Healthy aging did not show an effect on the heart rate (Figure 2A; 271.5 ± 11.7 vs. 292.3 ± 14.1 BPM for Aging-vs.-Control; *p* = 0.326). Aging resulted in increased RV peak pressures (Figure 2B; 26.8 ± 0.9 vs. 23.0 ± 0.9 mmHg for Aging-vs.-Control; *p* = 0.017), while showing a modest non-significant effect on end-diastolic pressures (Figure 2C; 1.9 ± 0.4 vs. 1.3 ± 0.1 mmHg for Aging-vs.-Control; *p* = 0.085). Effects of aging on the load-dependent measures of RV contractility and relaxation are shown in Figure 2D. Aging significantly increased $\frac{dp}{dt}$max (1,611.7 ± 90.5 vs. 1,063.8 ± 101.7 mmHg/s for Aging-vs.-Control; *p* = 0.004) but did not demonstrate any effects on $\frac{dp}{dt}$min (−823.9 ± 60.4 vs. −814.7 ± 85.5 mmHg/s for Aging-vs.-Control; *p* = 0.940). Increased contractility index was observed for the aging group (Figure 2E; 60.1 ± 2.2 vs. 45.8 ± 3.5 1/s for Aging-vs.-Control; *p* = 0.012), while the time-constant of RV relaxation (tau) remained unchanged (Figure 2F; 10.7 ± 1.6 vs. 9.9 ± 0.8 ms for Aging-vs.-Control; *p* = 0.595).

Healthy aging led to increased RVFW thickness (0.90 ± 0.05 vs. 0.65 ± 0.05 mm for Aging-vs.-Control; *p* = 0.001), while not affecting the Fulton index (0.26 ± 0.03 vs. 0.27 ± 0.01 mg/mg for Aging-vs.-Control; *p* = 0.140). Moreover, aging was associated with decreased RV and LV weight normalized to body weight ($\frac{\text{RV Weight}}{\text{Body Weight}}$: 0.05 ± 0.007% vs. 0.06 ± 0.003% for Aging-vs.-Control, *p* = 0.026; $\frac{\text{LV Weight}}{\text{Body Weight}}$: 0.18 ± 0.007% vs. 0.23 ± 0.009% for Aging-vs.-Control, *p* = 0.0005). Specimen-specific RVFW thickness and Fulton index measurements are reported in Supplementary Table 1.

### RVFW Biomechanical Properties

**Figure 3:**
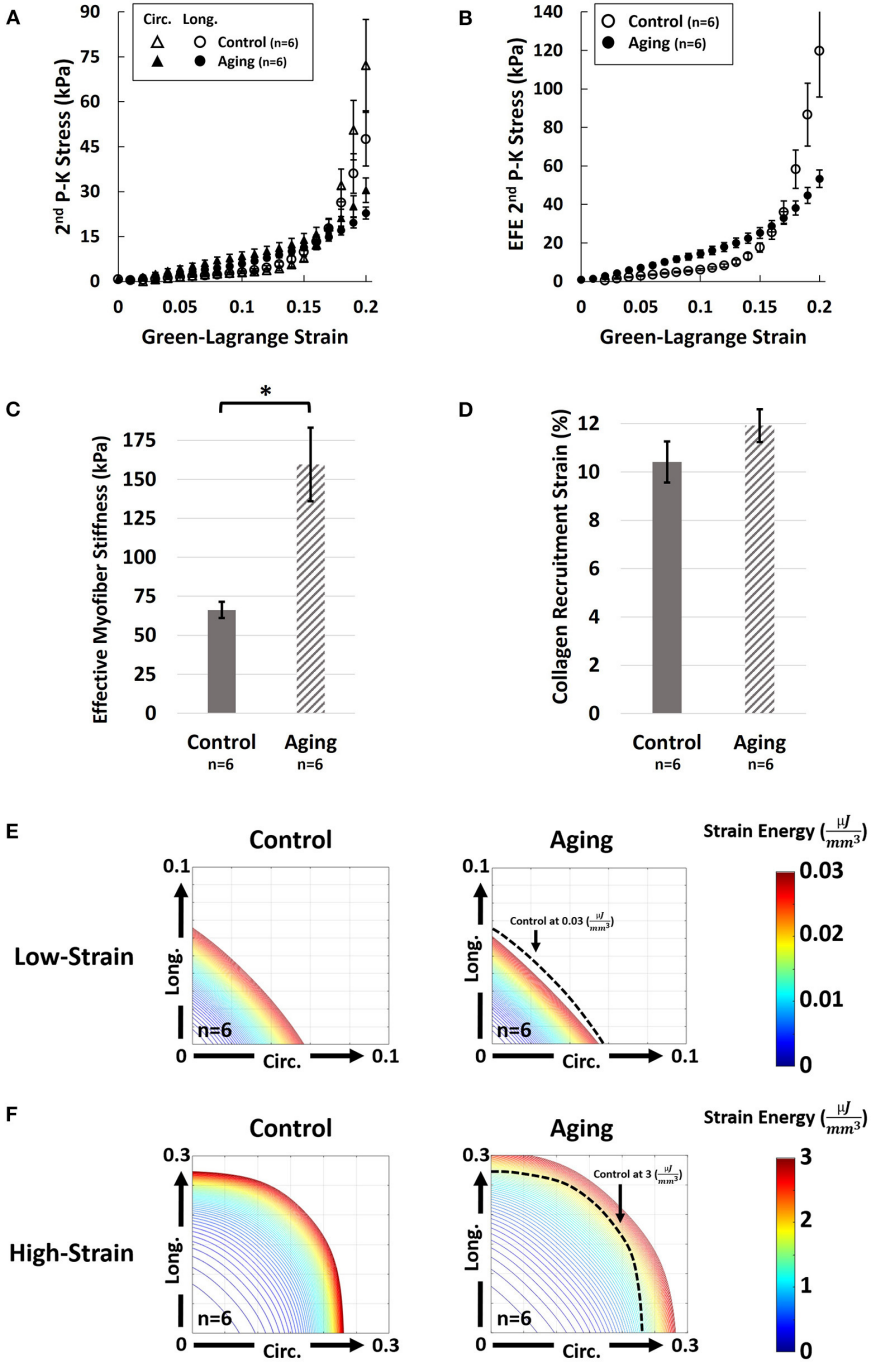
Effects of healthy aging on **(A)** Biaxial mechanical properties of RV myocardium, **(B)** Effective fiber-ensemble (EFE) mechanical properties of combined collagen-myofiber bundles, **(C)** Effective myofiber stiffness, **(D)** Collagen recruitment strain, **(E)** Strain energy maps of the RVFW in the low-strain region (circumferential-longitudinal strain space), and **(F)** Strain energy maps of the RVFW in the high-strain region. Healthy aging modulates the biomechanical properties of the RVFW in a bimodal manner by resulting in increased circumferential and longitudinal stiffness under lower strains, while progressing to decreased biaxial stiffness at higher strains. Significant myofiber stiffening was observed with healthy aging. Specimens in the aging cohort demonstrate higher levels of strain energy at equivalent levels of deformation compared to controls in the low strain region (indicating RVFW stiffening), while showing lower strain energy in the high-strain region (indicating a more compliant RVFW). Error bars represent standard error of the mean (SEM). ^*^Indicates *p* < 0.05. RV, Right ventricle; RVFW, Right ventricular free wall; 2nd P-K Stress, 2nd Piola-Kirchhoff stress; Circ, Circumferential; Long, Longitudinal; EFE 2nd P-K Stress, Effective fiber-ensemble 2nd Piola-Kirchhoff stress.

Aging demonstrated a bimodal effect on the RVFW biaxial properties by resulting in increased circumferential and longitudinal stiffness under lower strains, while progressing to decreased biaxial stiffness at higher strains (Figure 3A). A similar effect was observed on the EFE (effective fiber-ensemble) stress-strain properties of combined RVFW collagen-myofiber bundles (Figure 3B). Using a rule-of-mixtures approach, this translated into increased effective myofiber stiffness (Figure 3C; 159.5 ± 23.6 vs. 66.2 ± 5.2 kPa for Aging-vs.-Control; *p* = 0.003), while no significant effects were observed on collagen recruitment strain (Figure 3D; 11.9 ± 0.7% vs. 10.4 ± 0.9% for Aging-vs.-Control; *p* = 0.197). Specimen-specific constitutive model parameters for each group are shown in Supplementary Table 2. Overall, the employed model showed an acceptable fit quality (*R*^2^) to our experimental data (*R*^2^ = 0.95 ± 0.01 and 0.96 ± 0.01 for Aging and Control, respectively). Age-specific strain energy maps, representing the combined effects of all model parameters, are demonstrated in Figures 3E,F for each cohort at the low-strain and high-strain regions.

### Quantitative Transmural Histology

**Figure 4:**
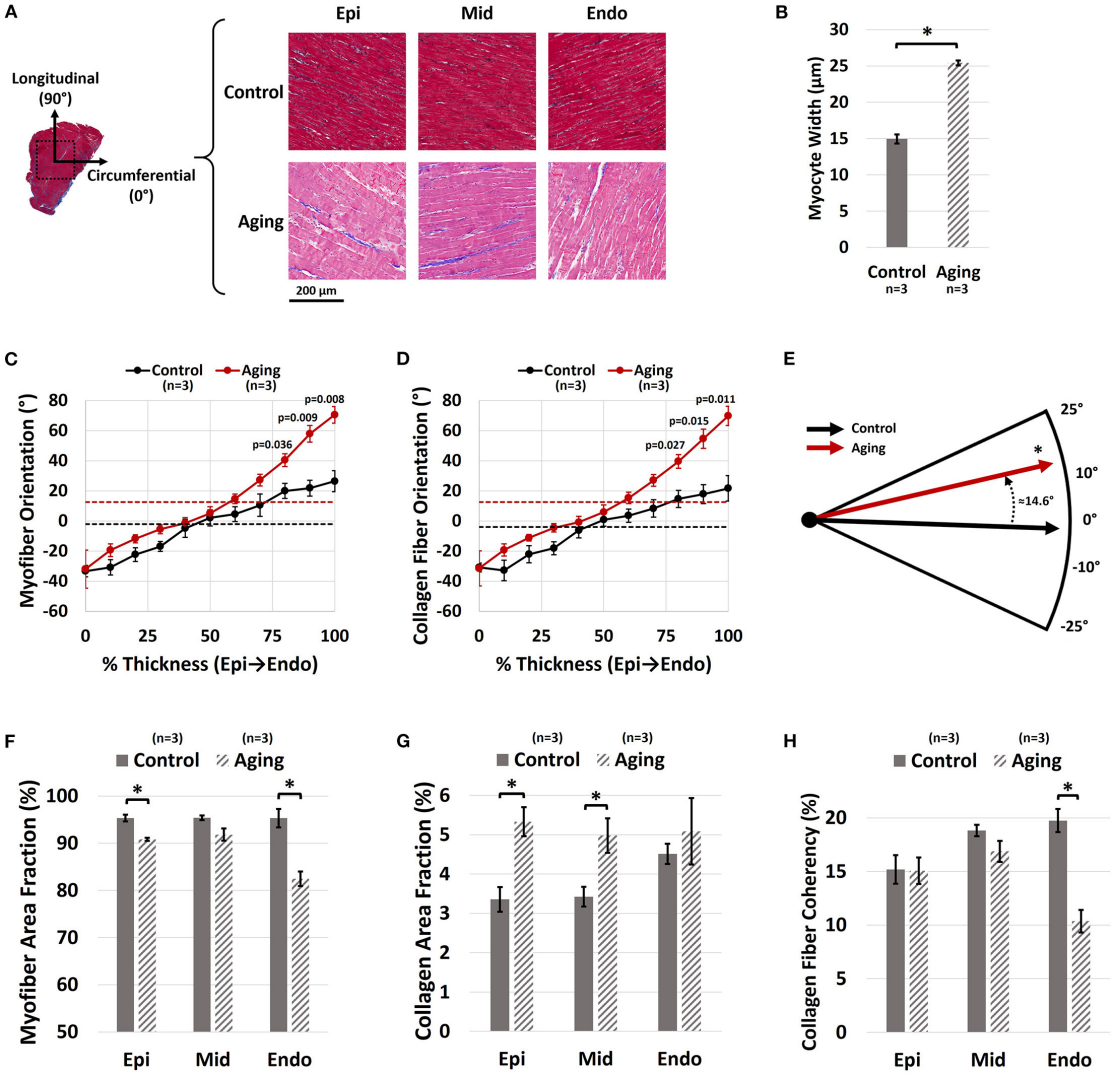
Histological analysis of the effects of healthy aging on RV structure. **(A)** Representative transmural histological sections of the RVFW (Red/Pink: Myofiber, Blue: Collagen) and effects of aging on **(B)** Cardiomyocyte hypertrophy (myocyte width), **(C)** Transmural myofiber orientations, **(D)** Transmural collagen fiber orientations, **(E)** Dominant myofiber orientations, **(F)** Transmural myofiber content (area fraction), **(G)** Transmural collagen content (area fraction), and **(H)** Transmural collagen fiber coherency. Healthy aging results in cardiomyocyte hypertrophy, in addition to reorientation of sub-endocardial collagen and myofibers toward the longitudinal direction. This is accompanied by cardiomyocyte loss, RVFW fibrosis, and decreased collagen fiber coherency. Error bars represent standard error of the mean (SEM). ^*^Indicates *p* < 0.05. RV, Right ventricle; RVFW, Right ventricular free wall; Epi, Epicardium; Mid, Mid-ventricular region; Endo, Endocardium.

Representative histological sections for each group are demonstrated in Figure 4A. Aging resulted in increased cardiomyocyte width (Figure 4B; 25.42 ± 0.34 vs. 14.94 ± 0.64 μm for Aging-vs.-Control; *p* = 0.0001). Quantifying the transmural orientation of RVFW fibers revealed myofiber (Figure 4C) and collagen (Figure 4D) reorientation toward the longitudinal direction at sub-endocardial levels. Overall, myofibers showed similar orientations to collagen fibers. Aging significantly shifted the overall orientation of myofibers (circular mean of transmural fiber angles, dotted lines in Figure 4C) by 14.6° toward the longitudinal direction (Figure 4E; *p* = 0.017). Similarly, the overall orientation of collagen fibers was shifted by 16.4° (*p* = 0.013). Aging also resulted in cardiomyocyte loss and decreased myofiber area fractions at both epicardium (Figure 4F; 90.8 ± 0.3% vs. 95.3 ± 0.7% for Aging-vs.-Control; *p* = 0.004) and endocardium (Figure 4F; 82.4 ± 1.5% vs. 95.3 ± 1.9% for Aging-vs.-Control; *p* = 0.007). Furthermore, aging led to RVFW fibrosis and increased collagen area fractions at epicardium (Figure 4G; 5.3 ± 0.4% vs. 3.4 ± 0.3% for Aging-vs.-Control; *p* = 0.015) and the mid-ventricular region (Figure 4G; 5.0 ± 0.4% vs. 3.4 ± 0.3% for Aging-vs.-Control; *p* = 0.037). Analyzing the coherency of collagen architectures revealed decreased coherency at the endocardium (Figure 4H; 10.4 ± 1.1% vs. 19.7 ± 1.1% for Aging-vs.-Control; *p* = 0.003), while showing no effects on the other regions.

## Discussion

In this pilot study of the effects of healthy aging on RV remodeling, we found aging associated with (1) increased RV peak pressures and contractility; (2) increased RVFW thickness in proportion to increased LV size; (3) longitudinal reorientation of collagen/myofibers, with transmural cardiomyocyte loss and RVFW fibrosis; and (4) increased effective myofiber stiffness. The increase in RV peak pressures (Figure 2B) is consistent with previous reports of increased PA pressures with healthy aging (9, 10). Increased PA pressures impose an increased afterload on the RV, leading to elevated RV pressures. Furthermore, cardiomyocyte width (Figure 4B) and RVFW thickness (Supplementary Table 1) increased with aging, leading to increased organ-level contractility (Figures 2D,E). Interestingly, these changes are similar to those seen in a PH model in young animals (18). Increased RVFW thickness was accompanied by reduced ratios of RV and LV weight normalized to body weight of the animals. This indicate RV growth with aging that is not proportional to the increase in body weight (reduced $\frac{RV\;Weight}{Body\;Weight}$ ratio), similar to previous reports of LV growth (41). Moreover, consistent with prior work (12, 42), reduced cardiomyocyte area fraction (Figure 4F) and RVFW fibrosis (Figure 4G) were noted with aging. Reduced cardiomyocyte area fraction in the RVFW increases the hemodynamic load on the remaining myocytes (43), possibly explaining the observed hypertrophy and stiffening patterns (Figures 4B, 3C).

Histological analyses revealed reorientation of endocardial collagen and myofibers, resulting in a longitudinal shift in dominant transmural fiber orientations (Figure 4E). Similar patterns of longitudinal fiber reorientation have been reported with PH (18, 34). Unlike PH, where elevated RV pressures may stimulate transmural fiber reorientation (20, 44), fiber realignment in aging may have different underlying mechanisms. A potential candidate, pending further investigation, is RV fiber reorientation due to volumetric growth of the RVFW with healthy aging (kinematic shift). Further analysis using growth-and-remodeling frameworks may facilitate decoupling the effects of growth-induced reorientation from fiber remodeling due to other mechanisms.

Fiber reorientation (Figures 4C,D) and increased transmural change in fiber angles with aging led to a less anisotropic biaxial mechanical response (Figure 3A) with bimodal alterations in RVFW biaxial properties (Figure 3). Specimens in the aging cohort demonstrated higher levels of strain energy at equivalent levels of deformation compared to controls in the low-strain region (indicating RVFW myofiber stiffening), while showing lower strain energy in the high-strain region (indicating more compliant collagen in the RVFW) (Figures 3E,F). Moreover, aging led to increased effective myofiber stiffness (Figure 3C). Potential underlying mechanisms of myofiber stiffening include myocyte remodeling due to cell loss, as well as reduced titin phosphorylation (45). Increased myofiber stiffness and reduced tissue-level ventricular stiffness at high strains have been previously documented in separate studies on age-related LV remodeling (46, 47). Despite alterations in tissue-level properties, the time-constant of RV relaxation (tau) did not show any changes with aging. It should be noted that, in addition to passive tissue properties, RV relaxation velocities can also affect tau and alterations in relaxation velocities have the potential to offset the changes in mechanical properties at the organ level. However, no direct measurement of relaxation velocities was performed in this work. Interestingly, we have previously observed myofiber stiffening to be accompanied by an increase in tau in response to PH in male Sprague-Dawley rats (19). A potential explanation for the observed differences with healthy aging compared to PH could be the severity of elevations in RV pressures and higher levels of hypertrophy in PH compared to aging-induced remodeling, that may manifest in concurrent changes in myofiber-level RV relaxation velocities and myocyte stiffening, affecting the time constant of RV relaxation at the organ-level. The underlying mechanisms of the observed effect warrant further cell and fiber-level investigation of aging-induced alterations in RV myocyte mechanics in future work.

No effects on the collagen recruitment strain (measure of collagen crimp) were observed with healthy aging (Figure 3D). However, aging led to RVFW fibrosis and increased collagen area fractions (Figure 4G). Despite an increased collagen content with similar levels of crimp to young controls, tissue-level stiffness of the specimens in the aging group was reduced in the high-strain region, when collagen fibers are recruited. This indicates a potential reduction in the intrinsic fiber-level stiffness of collagen fibers with aging. Additionally, reduced collagen fiber coherency was detected at the endocardial levels (Figure 4H), indicating a more sparse and isotropic distribution of collagen fibers (38). This has the potential to affect the load transfer mechanism of endocardial collagen, contributing to reduced stiffness at the tissue level. Ongoing research focuses on evaluation of lysyl oxidase-mediated alterations in collagen cross-linking with healthy aging, as a potential mechanism of reduced collagen network stiffness.

There are limitations to the experimental and modeling techniques used in this study. We only analyzed the effects of healthy aging in male animals. Previous work has shown sex-related differences in RV mechanics in PH (48–50), mainly due to the protective effects of the female sex hormone 17β-estradiol (estrogen). Recent studies on sex difference in RV-PA coupling in the setting of PH and heart failure with preserved ejection fraction have demonstrated superior adaptive remodeling in female patients leading to preserved RV-PA coupling at rest and under exercise, while male patients demonstrated impaired contractile function in response to increased afterload and lower RV-PA coupling (51, 52). While, the exact underlying mechanisms of the observed effects remain unknown (52) and, to the best of our knowledge, to date no data exist on sex-related differences in RV biomechanics with healthy aging, estrogen-mediated effects have the potential to affect the observed patterns in our work via altered adaptation in the myocyte contractile apparatus, potentially leading to different levels of structural and biomechanical remodeling. Sex-related difference in RV remodeling with healthy aging is an important topic necessitating further investigation in future studies. Moreover, as a first step toward better understanding of the effects of healthy aging on RV remodeling, the current work evaluated the changes in RV mechanics in the absence of analyzing LV structure/function and pulmonary hemodynamics. Due to the interdependence of RV and LV function (53), the observed effects may not be independent of potential age-related changes in LV hemodynamics or biomechanical properties. Biventricular analysis of the effects of healthy aging on RV and LV structure, function, and biomechanics, as well as pulmonary mechanics, is an important topic to be investigated in future research. Despite low variability and strong statistics, the small sample size of our pilot study limited our ability to investigate detailed interactions between aging, gender, and disease, which will require future studies with larger sample sizes. Furthermore, lack of molecular studies to evaluate the underlying mechanisms of the observed effects at the tissue and fiber level remains another limitation of the current work, requiring further investigation in future studies. We employed a phenomenological constitutive model for analyzing our biomechanical data; future work will focus on structurally-informed constitutive models of RV myocardium (21) to couple the histologically measured tissue architecture to biaxial properties. Different batches of staining solution used for each group resulted in different shades of cardiomyocyte staining for control vs. aging (red vs. pink). However, this had minimal effects on our findings as segmentation thresholds for myofibers and collagen were individually selected for each histological section. While, to the best of our knowledge, there has been no reports of biaxial testing-induced permanent alterations in soft tissue fiber architectures, lack of a dedicated group for histological analysis of the aging cohort remains a limitation of this work.

In summary, our results demonstrate that healthy aging may modulate RV remodeling via increased peak pressures, cardiomyocyte loss, fibrosis, fiber reorientation, and altered mechanical properties. While this can help our understanding of age-related changes in the cardiovascular fitness and response to disease, these findings need to be considered in light of potential sex-differences in RV remodeling and the limitations of the current work.

## Data Availability Statement

The raw data supporting the conclusions of this article will be made available by the authors, without undue reservation.

## Ethics Statement

The animal study was reviewed and approved by University of Pittsburgh Institutional Animal Care and Use Committee (IACUC).

## Author Contributions

DS: conception of the study, data acquisition, analysis and interpretation, and drafting the manuscript. YS: conception of the study, data acquisition, interpretation, and drafting the manuscript. TB: data analysis and interpretation and drafting the manuscript. EG, KK, and MS: conception of the study, data interpretation, and drafting the manuscript. All authors contributed to the article and approved the submitted version.

## Funding

This study was supported by the American Heart Association (AHA-20PRE35210429, DS; AHA Postdoctoral Fellowship 826806, YS) and the National Institutes of Health (NIH Grants 1R01AG058659, 2P01HL103455, and UL1 TR001857, MS; 2R01HL130261, 2R01HL113178, and R01HL150638, EG). The funding sources had no involvement in design of the study, data acquisition or interpretation.

## Conflict of Interest

MS: Research support from Aadi. Steering committee for Janssen. Consultancy fees from Acceleron and Bial. DS is employed by Align Technology, Inc. The remaining authors declare that the research was conducted in the absence of any commercial or financial relationships that could be construed as a potential conflict of interest.

## Publisher's Note

All claims expressed in this article are solely those of the authors and do not necessarily represent those of their affiliated organizations, or those of the publisher, the editors and the reviewers. Any product that may be evaluated in this article, or claim that may be made by its manufacturer, is not guaranteed or endorsed by the publisher.
