# Supplementary material for: The Effects of Healthy Aging on Right Ventricular Structure and Biomechanical Properties: A Pilot Study
Source: Front Med (Lausanne). 2022 Jan 10;8:751338. doi: 10.3389/fmed.2021.751338 (PMC8784691; doi:10.3389/fmed.2021.751338)
Supplement: Supplementary file 1 [file Data_Sheet_1.pdf]

Supplementary Material

1 Supplementary Figures

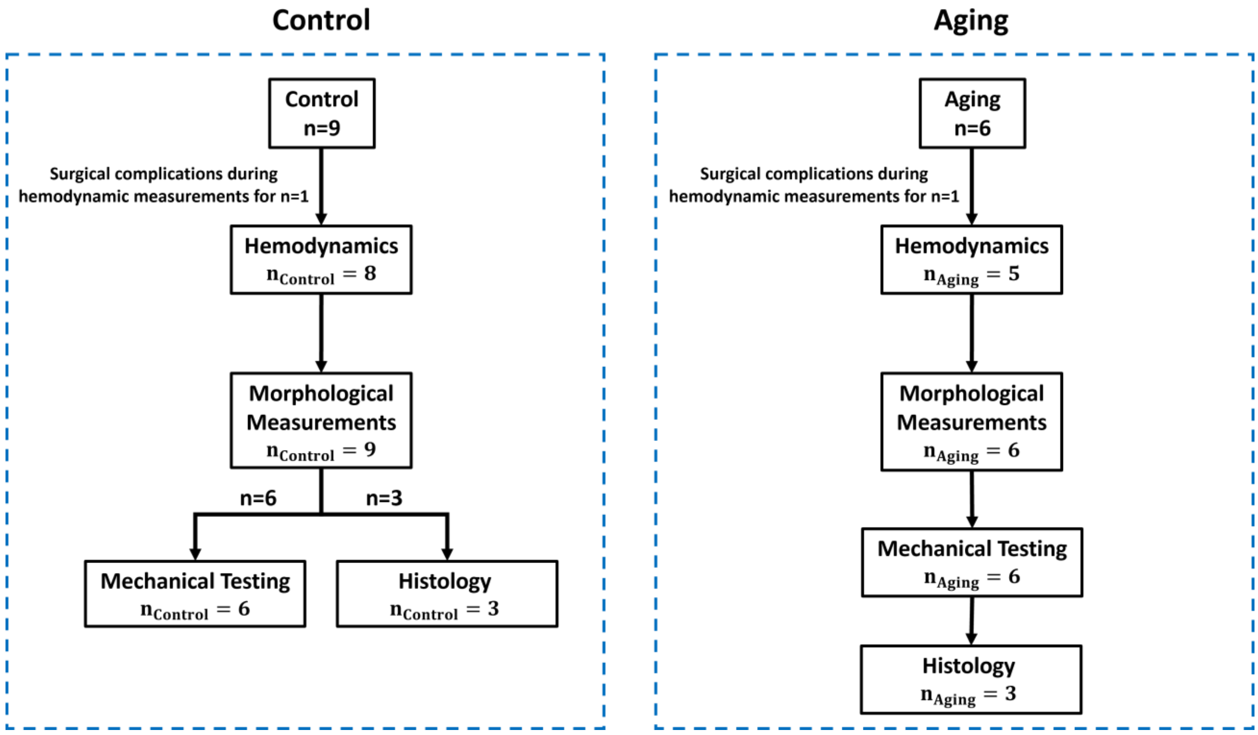

Supplementary Figure S1: Sample size and different biomechanical procedures performed on specimens from each group. In the current work, $n = 6$ animals were dedicated to each group to study the effects of aging on RV hemodynamics, morphology, and biomechanical properties. Histology for the aging cohort, was performed on a sub-set ($n = 3$) of the 6 animals used for hemodynamics and biomechanical analysis, mainly due to the limited availability of aging animals. However, in the control group we were able to have 3 separate animals dedicated to histological analysis of fiber architecture and tissue content, in addition to the 6 animals used for biomechanical testing. Hemodynamic and morphological measurements were still performed on these 3 additional control animals, in order to confirm normal RV function.

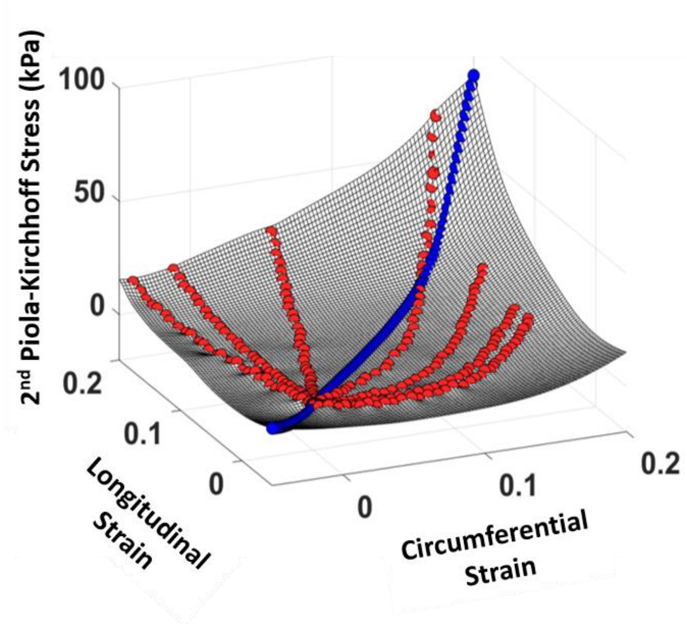

Supplementary Figure S2: Representative demonstration of interpolation of RVFW stress response under equibiaxial strain-controlled loading (blue) using the scattered multi-protocol stress-strain measurements (red; 1:1, 1:2, 2:1, 1:4, 4:1, 1:6, and 6:1 displacement-controlled loading scenarios). Please note that the demonstrated interpolation shows the stress estimations for one component of the biaxial RVFW stress response. Similar interpolations were reiterated for estimations in the other direction.

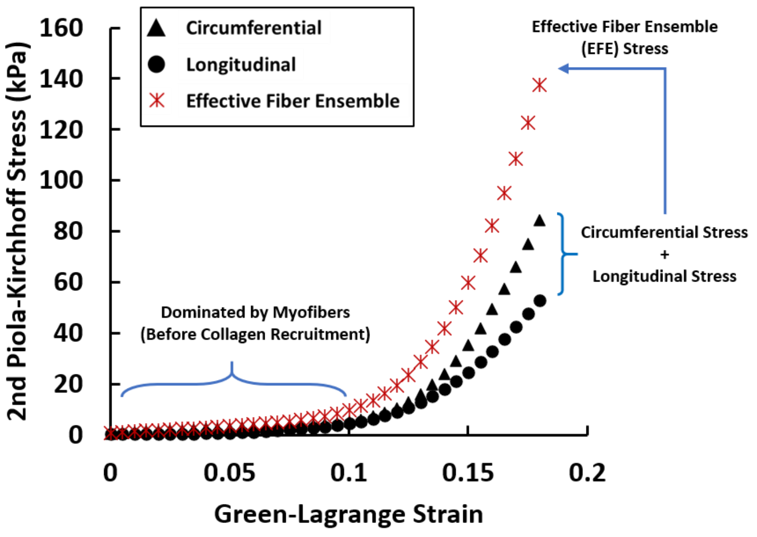

Supplementary Figure S3: Representative demonstration of approximation of the effective fiber-ensemble (EFE) stress-strain response based on equibiaxial tissue-level properties.

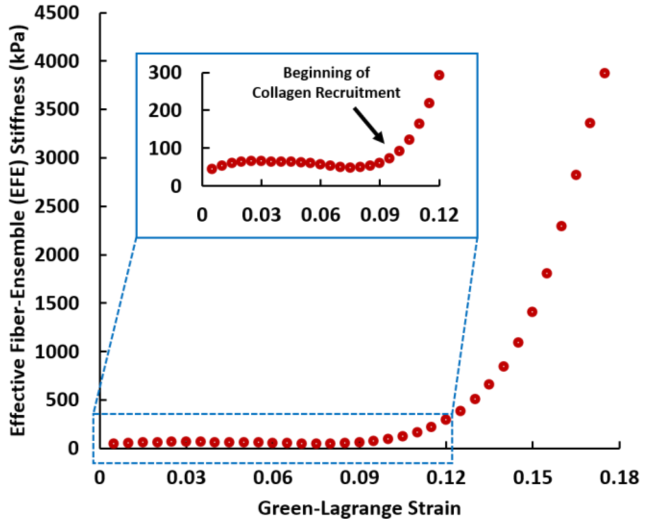

Supplementary Figure S4: Representative demonstration of differentiation of the effective fiber-ensemble (EFE) stress response with respect to strain, for estimation of EFE stiffness and identification of collagen recruitment strain.

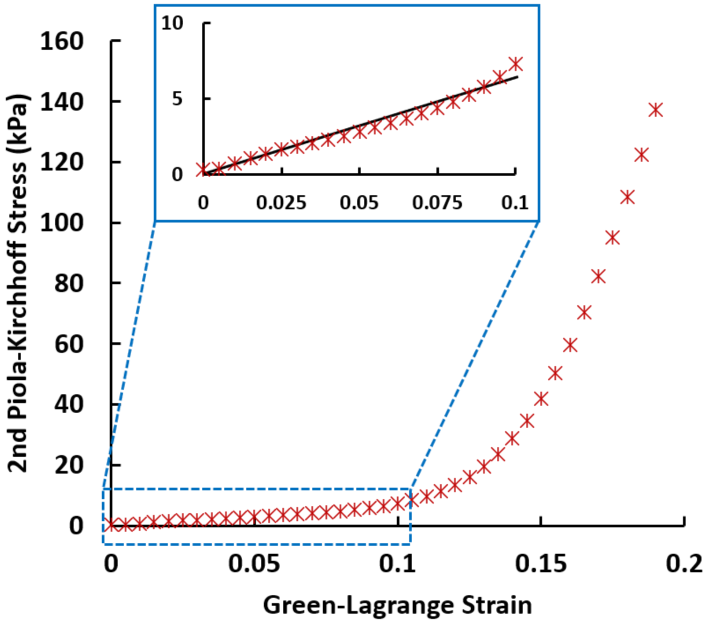

Supplementary Figure S5: Representative demonstration of linear regression of EFE properties in the low-strain region, for characterization of $(\mathrm{TM}_{\mathrm{EFE}})_{\mathrm{Before\ Collagen\ Recruitment}}$ and estimation of effective myofiber stiffness

2 Supplementary Tables

Supplementary Table S1: Effects of healthy aging on RV hypertrophy (RVFW thickness), Fulton index, and normalized ratios of RV and LV weight to body weight

		**RVFW Thickness (mm)**	**Fulton Index (mg/mg)**	$\frac{\text{RV Weight}}{\text{Body Weight}}$ (%)	$\frac{\text{LV Weight}}{\text{Body Weight}}$ (%)
**Control (n=9)**	**Specimen#1**	0.65	0.25	0.06	0.23
	**Specimen#2**	0.75	0.23	0.05	0.22
	**Specimen#3**	0.75	0.27	0.06	0.23
	**Specimen#4**	0.55	0.29	0.06	0.20
	**Specimen#5**	0.70	0.29	0.06	0.21
	**Specimen#6**	0.55	0.28	0.06	0.21
	**Specimen#7**	0.65	0.26	0.07	0.27
	**Specimen#8**	0.65	0.28	0.08	0.27
	**Specimen#9**	0.65	0.29	0.08	0.26
	**Average**	**0.65±0.05**	**0.27±0.01**	**0.06±0.003**	**0.23±0.009**
**Aging (n=6)**	**Specimen#1**	1.05	0.38	0.08	0.20
	**Specimen#2**	0.85	0.23	0.04	0.17
	**Specimen#3**	0.90	0.23	0.04	0.17
	**Specimen#4**	0.80	0.22	0.04	0.19
	**Specimen#5**	0.70	0.22	0.03	0.15
	**Specimen#6**	1.05	0.28	0.05	0.17
	**Average**	**0.90±0.05**	**0.26±0.03**	**0.05±0.007**	**0.18±0.007**
**p Value**		0.001	0.140	0.026	<0.001

Supplementary Table S2: Constitutive model parameters representing the circumferential (b_1), longitudinal (b_2) and in-plane coupling (b_3) stiffness of RV myocardium for specimens in the control and aging groups

		Model Parameters				Goodness of Fit (R^2)
		**B_0 (kPa)**	**b_1**	**b_2**	**b_3**	
**Control (n=6)**	**Specimen#1**	0.25	122.20	63.32	63.03	0.93
	**Specimen#2**	0.21	61.66	48.69	37.15	0.97
	**Specimen#3**	0.12	62.21	90.45	72.56	0.95
	**Specimen#4**	0.21	76.50	77.09	66.29	0.96
	**Specimen#5**	0.13	124.19	78.37	78.21	0.98
	**Specimen#6**	0.12	142.59	100.03	83.09	0.95
**Aging (n=6)**	**Specimen#1**	0.17	86.02	50.85	54.42	0.93
	**Specimen#2**	0.46	42.12	32.12	31.65	0.96
	**Specimen#3**	0.30	65.50	50.28	46.37	0.94
	**Specimen#4**	0.44	54.91	46.73	36.12	0.98
	**Specimen#5**	0.68	39.63	28.36	38.08	0.95
	**Specimen#6**	0.16	47.91	75.73	52.61	0.94
